# Erratum in: MicroRNA-124 Dysregulation Is Associated With Retinal Inflammation and Photoreceptor Death in the Degenerating Retina

**DOI:** 10.1167/iovs.66.13.28

**Published:** 2025-10-16

##  

***Erratum in:*** “MicroRNA-124 Dysregulation Is Associated With Retinal Inflammation and Photoreceptor Death in the Degenerating Retina” by Joshua A. Chu-Tan, Matt Rutar, Kartik Saxena, Riemke Aggio-Bruce, Rohan W. Essex, Krisztina Valter, Haihan Jiao, Nilisha Fernando, Yvette Wooff, Michele C. Madigan, Jan Provis, and Riccardo Natoli (*Invest Ophthalmol Vis Sci*. 2018;59(10):4094–4105), https://doi.org/10.1167/iovs.18-24623.

The images and data pertaining to *Ccl2* mRNA expression in dim-reared versus PD animals (Figs. 5H and 5I) were derived from retinal sections from a previous study, which we failed to cite. To illustrate the findings, the authors incorrectly selected an image (Fig. 5H) from a control IgG animal, rather than one where photoreceptor death was the only treatment, and which is a partial duplication of Figure 3b of Natoli R, Fernando N, Madigan M, et al. Microglia-derived IL-1β promotes chemokine expression by Müller cells and RPE in focal retinal degeneration. *Mol Neurodegener*. 2017;12(1):31. DOI: 10.1186/s13024-017-0175-y. This error has no impact on the results or conclusions of this study. The figure legend also incorrectly stated *n* = 4, when the figure clearly indicates *n* = 3. Figure 5 has now been replaced with a revised version in the article online, and the legend has been corrected.

**Original Figure 5.**
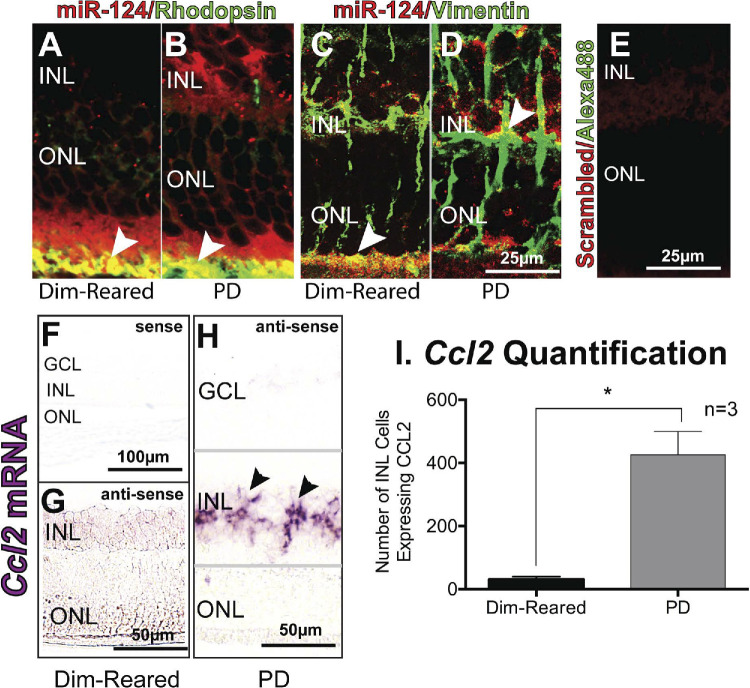
Colocalization of miR-124 with photoreceptors and Müller cells. (**A, B**) Immunohistochemistry using a photoreceptor cell marker, rhodopsin (*green*), showed colocalization with miR-124 staining beneath the ONL (*arrowheads, yellow*) in both dim-reared and PD rodents. (**C, D**) Immunohistochemistry with Müller cell marker, vimentin (*green*), also showed colocalization with miR-124 labeling, both in the OLM for dim-reared rodents and in the INL for PD rodents (*arrowheads, yellow*). (**E**) Scrambled control showed no red or green staining. (**F–H**) In situ hybridization for *Ccl2* in rodents shows localization in the INL after PD when compared to dim-reared animals. (**I**) Quantification of *Ccl2*-expressing cell numbers in the INL showing positive staining revealed a significant increase. *Significance using a Student's *t*-test, *P* < 0.05, and error bars indicate SEM, *n* = 4.

**Corrected Figure 5.**
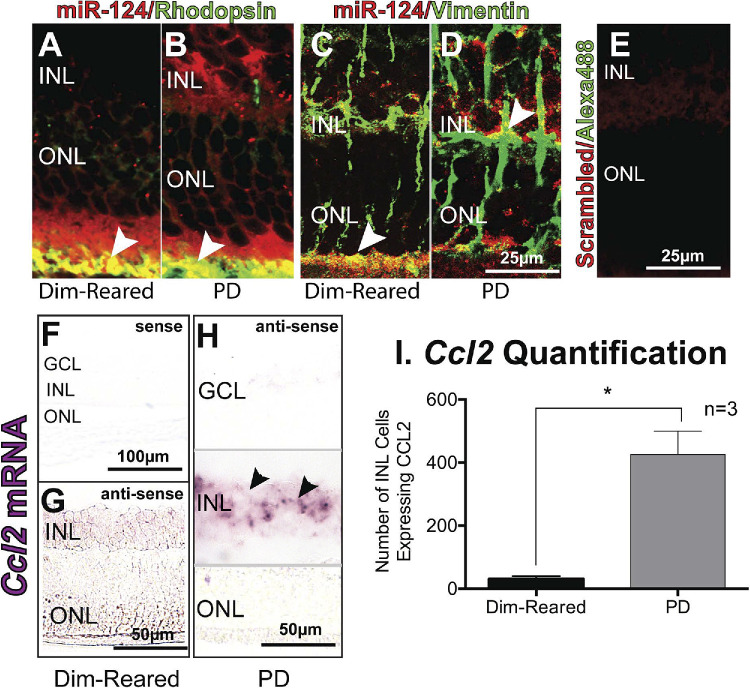
Colocalization of miR-124 with photoreceptors and Müller cells. (**A, B**) Immunohistochemistry using a photoreceptor specific marker, rhodopsin (*green*), showed colocalization with miR-124 staining (*red*) deep to the ONL (*arrowheads, yellow*) in both dim-reared and PD rodents. (**C, D**) Immunohistochemistry with the Müller cell marker, vimentin (*green*), also showed colocalization with miR-124 (*red*) in the deep ONL for dim-reared rodents, and in the INL for PD rodents (*arrowheads, yellow*). (**E**) No labeling was seen in the scrambled control. (**F–H**) In situ hybridization for *Ccl2* shows increased expression in the INL in PD rodents, compared to dim-reared (data from Natoli R, Fernando N, Madigan M, et al. Microglia-derived IL-1β promotes chemokine expression by Müller cells and RPE in focal retinal degeneration. *Mol Neurodegener*. 2017;12(1):31.) (**I**) Quantification of *Ccl2*-expressing cells in the INL of dim-reared vs PD rodents demonstrates a significant difference. *Student *t*-test, *P* < 0.05; error bars indicate SEM.

